# Rho-GTPases subfamily: cellular defectors orchestrating viral infection

**DOI:** 10.1186/s11658-025-00722-w

**Published:** 2025-05-02

**Authors:** Beibei Zhang, Shuli Li, Juntao Ding, Jingxia Guo, Zhenghai Ma, Hong Duan

**Affiliations:** 1 Xinjiang Key Laboratory of Biological Resources and Genetic Engineering, College of Life Science and Technology, Xinjiang University, Urumqi, Xinjiang China; 2 Disease Prevention and Control Center of Xinjiang Production and Construction Corps, Urumqi, Xinjiang China; 3 College of Veterinary Medicine, Henan Agricultural University, Zhengzhou, Henan China

**Keywords:** Rho-GTPases, Molecule switch, Viral infection, Regulatory mechanism, Therapeutic target

## Abstract

Ras homolog gene family-guanosine triphosphatases (Rho-GTPases), key molecular switches regulating cytoskeletal dynamics and cellular signaling, play a pivotal role in viral infections by modulating critical processes such as viral entry, replication, and release. This review elucidates the intricate mechanisms through which Rho-GTPases, via interactions with guanine nucleotide exchange factors (GEFs), GTPase-activating proteins (GAPs), and other signaling pathways, including the phosphoinositide 3-kinase/protein kinase B (PI3K/Akt), rat sarcoma (Ras), and nuclear factor kappa-light-chain-enhancer of activated B cells (NF-κB) pathways, facilitate viral pathogenesis. Specific viruses, such as influenza A virus (IAV), herpesviruses, human immunodeficiency virus (HIV), and respiratory syncytial virus (RSV), exploit Rho-GTPase-mediated cytoskeletal reorganization to enhance infectivity. For example, Rho-GTPases promote actin remodeling and membrane fusion, which are essential for viral entry and intracellular transport. Furthermore, Rho-GTPases modulate immune responses, often suppressing antiviral defenses to favor viral replication. Despite these insights, the molecular mechanisms underlying Rho-GTPase regulation during viral infections remain incompletely understood. Future research should focus on delineating the precise roles of Rho-GTPases in distinct viral life cycles, uncovering novel regulatory mechanisms, and developing targeted antiviral therapies that selectively inhibit Rho-GTPase signaling without compromising host cell functions. Such advancements could pave the way for broad-spectrum antiviral strategies, particularly against viruses that heavily rely on cytoskeletal manipulation for infection.

## Introduction

Rho-GTPases, a subfamily of the small G protein superfamily, play a pivotal role in diverse physiological processes, including cell cytoskeleton remodeling (regulating cell shape, movement, and migration), signal transduction (mediating cell differentiation, proliferation, and apoptosis), and physiological conditions such as tumorigenesis (facilitating malignant tumor occurrence, invasion, and metastasis). These functions are critical in both normal cellular physiology and disease states [1]. Notably, viruses exploit the cytoskeletal dynamic changes and host immune responses by hijacking Rho-GTPase-mediated signaling pathways during infection. Specifically, during viral attachment, entry, and egress, the cytoskeleton—a fundamental structural framework of the cell—is manipulated by viruses to overcome physical barriers, enabling intracellular trafficking and completion of the viral life cycle. In these processes, Rho-GTPases act as molecular switches, ensuring precise localization of viral genetic material for transcription and translation, as well as facilitating the assembly of viral structural components derived from multiple organelles into mature virions. These virions are subsequently disseminated via extracellular release or cell-to-cell transmission. Conversely, Rho-GTPase-mediated signaling pathways also modulate immune responses by modulating cell morphology and cytoskeletal dynamics, which in turn influence key immunological processes such as antigen recognition, immune cell activation, and effector functions, ultimately contributing to the regulation of immune clearance and memory [2]. This dual role enhances the specificity and efficiency of immune responses while simultaneously being exploited by viruses to promote infection. Given the central role of Rho-GTPases in viral pathogenesis, targeted disruption of Rho-GTPase-mediated pathways represents a promising strategy to prevent viral hijacking of host biosynthetic metabolic processes, thereby restoring normal cellular physiology and inhibiting infection [3–5].

The Rho-GTPases superfamily comprises approximately 20 members, which are classified into five distinct subfamilies on the basis of structural and functional characteristics: the Rho-GTPase subfamily, the Ras-related C3 botulinum toxin substrate (Rac) subfamily, the cell division cycle 42 (Cdc42) subfamily, the resistance-nodulation-division (Rnd) subfamily, and the Rho-related BTB domain-containing protein 1 (Rho BTB) subfamily [6]. Members of the Rho-GTPase subfamily, including RhoA, RhoB, and RhoC, exhibit high sequence homology and are ubiquitously expressed across various cell types. These proteins are primarily responsible for the formation of actin stress fibers and the aggregation of focal adhesion complexes, thereby maintaining cellular cytoskeletal shape [7]. The Rac subfamily, encompassing Rac1, Rac2, and Rac3, plays a key role in promoting lamellipodia and membrane ruffling, processes essential for cell migration, adhesion, and immune responses [8–10]. The Cdc42 subfamily, which includes Cdc42, T-cell lymphoma invasion and metastasis-inducing protein 10 (TC10), and T-cell leukemia/lymphoma protein (TCL), is central to cytoskeletal dynamics [11]. Cdc42, the core member of this subfamily, activates downstream effectors such as Wiskott–Aldrich syndrome protein (WASP), myotonic dystrophy kinase-related Cdc42-binding kinase (MRCK), and p21-activated kinase 1 (PAK1) to regulate actin polymerization and depolymerization, facilitating the reorganization of the cytoskeleton and the formation of cellular protrusions (e.g., filopodia, lamellipodia, and stress fibers) [12, 13]. Additionally, Cdc42 is implicated in critical physiological processes, including cell division, apoptosis, and cycle regulation [14, 15]. The Rnd subfamily, comprising Rnd1, Rnd3/RhoE, and Rnd2, plays a significant role in cytoskeletal regulation [16]. Although Rnd proteins share functional overlap with Rho proteins, their interaction mechanisms differ owing to their integration within intracellular signaling network [11]. Furthermore, Rnd proteins often exhibit antagonistic or regulatory effects on Rho-mediated signaling pathways [17]. The RhoBTB subfamily, including RhoBTB1 and 2, is less well characterized; however, emerging evidence suggests their involvement in signal transduction, gene expression regulation, and protein degradation, mediated by their BTB domains, which facilitate protein–protein interaction [18, 19].

Emerging evidence highlights the critical role of Rho-GTPase superfamily members in facilitating viral dissemination and transmission. These proteins interact with diverse signaling molecules to regulate cytoskeletal dynamics, cell polarity, and growth-related pathways, while also modulating host cell recognition and responses to viral infection. Such mechanisms are intricately linked to viral pathogenesis and host–pathogen interactions [2, 4, 20]. However, the molecular underpinnings of these processes, particularly the crosstalk between Rho-GTPases and other signaling pathways, remain poorly understood. Elucidating these mechanisms is essential, as they hold significant implications for understanding Rho-GTPase-mediated viral infections, advancing antiviral research, and developing targeted therapeutic strategies. Future studies should prioritize unraveling these complex interactions to identify novel antiviral targets and improve clinical outcomes.

## Correlation between Rho-GTPases and viral infection

**Fig. 1:**
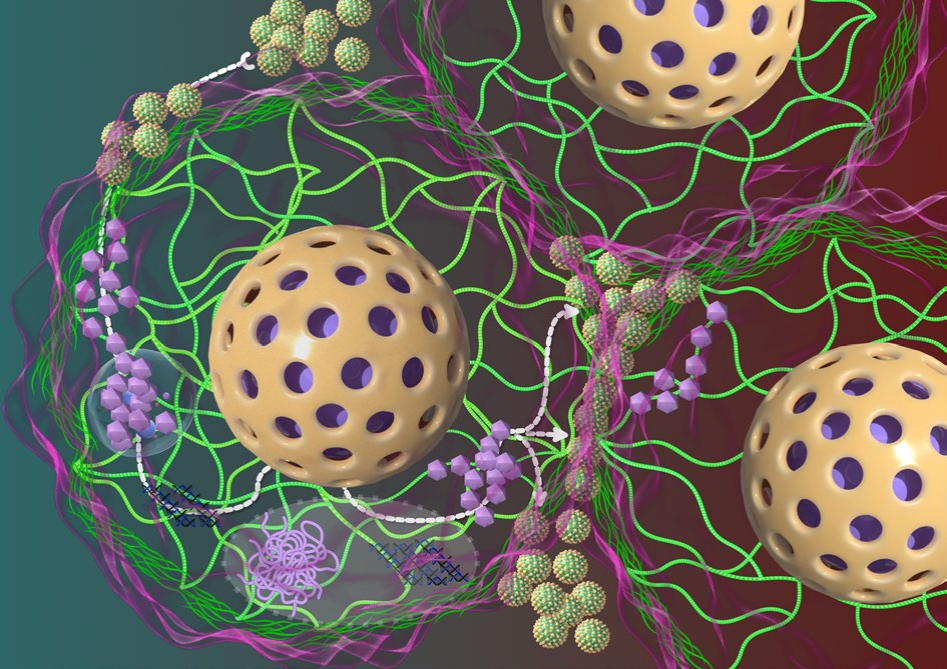
An illustration of the pivotal role of Rho-cytoskeleton dynamics in viral infection processes. The Rho-GTP/Rho-GDP cycle serves as a critical regulatory hub, orchestrating cytoskeletal reorganization to maintain cellular morphology and structural integrity while facilitating essential physiological functions including intracellular trafficking, signal transduction, and cell division. During viral infection, this intricate cytoskeletal network undergoes dynamic remodeling through Rho protein activation, particularly in processes involving membrane deformation and intracellular transport mechanisms. The viral life cycle is fundamentally dependent on the coordinated interaction between viral particles, membrane receptors, and the Rho-cytoskeleton system. This tripartite interaction enables efficient viral entry into host cells, followed by precisely regulated intracellular transport. Subsequent viral uncoating and nucleic acid release into the cytoplasm or nucleus facilitate the production of viral transcription and translation products. These components are ultimately assembled into progeny virions, which are subsequently released for intercellular dissemination. Of particular significance is the cytoskeleton’s dual role in these processes, providing both the mechanical forces necessary for viral transport and the structural framework for intracellular movement. The Rho-mediated cytoskeletal reorganization not only supports viral replication but also contributes to the spatial organization of viral components within the host cell, ensuring efficient viral production and propagation

Viral infection is a complex, multistep process involving intricate interaction between viruses and host cells. Central to this process is the virus’s ability to enter host cell via specific receptors and pathways, hijack host cellular resources for replication, and assemble new viral particles, ultimately leading to their release and the initiation of new infection cycles (Fig. 1). These steps are tightly regulated by host and viral factors [21]. The infection cycle begins with viral attachment to host cell surface receptors, often mediated by adhesion proteins and glycoproteins, followed by entry through endocytosis or membrane fusion [22]. Once inside, the virus releases its genetic material (DNA or RNA) through the combined activity of viral and host cell enzymes, enabling replication, transcription, and translation [23]. The newly synthesized viral components are then assembled into progeny viruses, which are released into the extracellular space via host cell lysis, budding, or membrane fusion, thereby perpetuating the infection cycle [24]. This exploitation of host machinery often disrupts normal cellular functions, leading to structural damage, functional impairment, and potentially cell death [25].

Rho-GTPases, key regulatory of cytoskeletal dynamics, contribute to virus infection by modulating processes critical for viral entry, replication, and dissemination. These small GTPases influence cytoskeletal reorganization, creating favorable conditions for viral attachment, intracellular transport, and egress [26, 27]. For example, viruses such as dengue virus (DENV) [28], pseudorabies virus (PRV) [29], and rabies virus (RABV) [30] exploit Rho-GTPases signaling to remodel the host cytoskeleton, facilitating viral invasion and intracellular trafficking. Beyond cytoskeletal regulation, Rho-GTPases also modulate host immune responses, including innate and adaptive immunity, thereby shaping the progression of viral infections. For instance, Rho-GTPases indirectly influences the cell cycle by regulating cell cycle regulators such as cyclin-dependent kinase 4 inhibitor D1 (cyclin D1) [31], negative regulatory factors such as cyclin-dependent kinase (CDK) [32], and cyclin-dependent kinase inhibitor 27 protein/cyclin-dependent kinase inhibitor (P27KiP1) [33], creating an environment conducive to viral replication. Furthermore, the Rho-GTPases is widely distributed in immune cells and indirectly promotes viral infection by regulating immune responses, innate immunity, and adaptive immunity. A canonical antiviral protein, mitochondrial antiviral-signaling protein (MAVS), which orchestrates the host innate immune response to RNA virus infection, has been found to interact with Rac1 during viral infection. This interaction restricts the association of MAVS with the E3 ubiquitin ligase tripartite motif-containing protein 31 (Trim31), thereby inhibiting MAVS ubiquitination, aggregation, and activation. Furthermore, Rac1 facilitates the recruitment of cysteine-dependent aspartate-specific protease-8 (caspase-8) and cellular FLICE-like inhibitory protein (cFLIP) to the MAVS signalosome, contributing to the failure of Receptor-interacting serine/threonine-protein kinase (Ripk1) cleavage and subsequent termination of MAVS signaling [34]. In summary, Rho-GTPases serve as central players in viral pathogenesis by orchestrating cytoskeletal dynamics, cell cycle regulation, and immune modulation. Their involvement in these processes not only facilitates viral replication and spread but also highlights their potential as therapeutic targets. Understanding the molecular mechanisms by which Rho-GTPases contribute to viral infections could pave the way for the development of novel antiviral strategies, offering significant implications for public health and the treatment of viral diseases.

## Basic mechanisms of Rho-GTPases in viral infection

### Activation and signaling of Rho-GTPases

Structurally, most members of the Rho-GTPases family possess lipid modifications at their C-terminus, enabling membrane anchoring—a feature critical for their localization and function. Like other small GTPases, Rho-GTPases cycle between an inactive guanosine diphosphate (GDP)-bound state and active GTP-bound state [35], a transition essential for their role as molecular switches. This cycling is tightly regulated by GEFs, which promote GDP dissociation and GTP binding to activate Rho-GTPases, and GAPs, which enhance GTP hydrolysis to return inactive Rho-GDP state. This dynamic regulation ensures precise control over Rho-GTPase activity, which is crucial for maintaining cellular homeostasis and responding to extracellular signals [1, 36]. Dysregulation of this process is implicated in various diseases, making Rho-GTPases and their regulatory proteins attractive therapeutic targets.

Functionally, Rho-GTPase act as molecular switch that control downstream signaling pathways by toggling between their active (GTP-bound) and inactive (GDP-bound) states [37]. In their active form, Rho-GTPases interact with cytoskeletal proteins such as actin and microtubules (MTs), regulating cytoskeletal assembly and disassembly. This dynamic regulation of the cytoskeleton is essential for processes such as cell motility, morphology, and intracellular transport. Notably, aberrant Rho-GTPase activity is closely associated with cancer progression, particularly in highly metastatic tumors. In this context, the hyperactivation of Rho-GTPase induces cytoskeletal rearrangement, leading to reduced cell adhesion and promoting tumor cell detachment and metastasis. Consequently, cells are more prone to detach from their original sites and undergo systemic dissemination, leading to a significant increase in patient mortality [38]. Conversely, inhibition of Rho-GTPase signaling can disrupt cytoskeletal dynamics, impairing cell movement and invasion [39]. Undoubtedly, multiple key determinants—including but not limited to extracellular matrix (ECM) remodeling [40], differential expression of microRNAs [41], and genomic instability [42]—have been extensively demonstrated to play essential roles in this biological process. From a mechanistic perspective, the vast majority of these intracellular participate, either directly or indirectly, in the intricate regulatory network governing cytoskeletal dynamics [43]. Moreover, in the process of signal transduction, the Rho-GTPases interacts with other signaling molecules to collectively regulate cell signal transduction and cellular function. Mechanistically, Rho-GTPases function as molecular switches by modulating actin polymerization and microtubule organization, enabling cell motility and morphological changes. Through crosstalk with major signaling cascades, they integrate extracellular cues with intracellular responses [44, 45]. In cancer, aberrant Rho signaling promotes tumor progression by enhancing proliferation, survival, invasion, and metastasis, making them crucial players in oncogenic transformation [43]. These interconnected mechanisms allow Rho-GTPases to coordinate complex cellular behaviors essential for development and homeostasis.

### Rho-GTPase-dependent cytoskeletal modulation in viral infection

Having established the critical role of Rho proteins in cytoskeletal regulation during viral infection, we now focus on the upstream mechanisms governing Rho activation. The pivotal role of the Rho-GTPases in viral infection lies primarily in their regulation of the cytoskeleton, which indirectly influences the infection process [46]. The cytoskeleton, composed of microfilaments, MTs, and intermediate filaments, not only maintains cellular morphology and internal structure but also facilitates essential cellular processes, such as motility, material transport, and signal transduction [47]. Virus infection typically involves interaction with the cell membrane, endocytosis of viral particle, intracellular transport, and release, all of which are closely linked to cytoskeleton dynamics [48, 49]. Rho-GTPases regulate the cytoskeletal reorganization by activating or inhibiting downstream effectors, including Rho-associated kinase (ROCK) [50] and mammalian Diaphanous (mDia)-related formin [51], which modulate the assembly and disassembly of actin filaments and MTs. Specifically, RhoA is primarily involved in stress fibers formation and focal adhesions assembly [52], while Rac1 and Cdc42 regulate leading-edge protrusions in migrating cells [53]. As a fundamental structural and functional framework, the cytoskeleton not only maintains cell shape and internal organization but also supports critical biological activities [54]. Among cytoskeletal regulators, Rho-GTPases are indispensable for viral infection, underscoring their significance in this context.

During the initial stages of viral infection, the virus must recognize and bind to specific receptors on the host cell surface, a process influenced by the cytoskeleton network. Rho-GTPases regulate cytoskeletal reorganization, thereby modulating the distribution and abundance of cell surface receptors and affecting viral recognition and binding [55]. For example, RhoA controls the actin filament dynamics, influencing receptor distribution and virus–cell interactions [56]. Following receptor attachment, enveloped viruses often enter cells via endocytosis, a process facilitated by Rho-GTPases through the regulation of vesicle formation, transport, and fusion [57]. Inside the cell, cytoskeletal dynamics further influence the release of viral genomes into the cytoplasm [58]. Rho-GTPases, particularly through downstream molecular processes such as Diaphanous homolog 2 (Dia-2), regulate vesicle maturation and fusion, ensuring efficient viral genome release [4]. During viral genome replication and viral protein synthesis, the cytoskeleton provides essential transport channels, with Rac1 and Cdc42 playing key roles in these processes [59]. Concurrently, RhoA activates ROCK to induce cytoskeletal contraction and reshaping, promoting tight junction formation and facilitating viral replication and assembly [60]. Finally, newly assembled viral particles are transported to the cell membrane via the cytoskeleton and released into the extracellular environment. It is evident that Rho proteins also participate in the late stage of viral life cycle by leveraging the cytoskeleton and closely collaborating with viral proteins. A notable example is the infection of CD4^+^ T lymphocytes by HIV, where the activation of Rac1, Cdc42, and RhoA depends on the membrane localization of the viral protein Gag and its interaction with specific membrane phospholipids. This interplay collectively mobilizes Rho family members to engage cytoskeletal signaling pathways, which in turn promotes further Gag membrane localization, ultimately facilitating membrane contraction and fusion to ensure efficient viral particle release [61].

## Regulatory mechanism of Rho-GTPases in viral infection

### Upstream regulatory networks of Rho-GTPases activation

Rho-GTPases serves as critical regulators of cell cytoskeleton dynamics, directly influencing various physiological functions through their active state. The activity of the Rho-GTPases is precisely regulated by upstream activators (GEFs) and inhibitors (GAPs), which initiate downstream signaling pathways through direct or indirect mechanisms [36, 62]. Current research has identified multiple GEF families with distinct specificities for different Rho-GTPases. For instance, the recombinant T-cell lymphoma invasion and metastasis-inducing protein 1 (Tiam) and Vav 1/2 oncogene (Vav1/Vav2) specifically target Rac [63, 64], while Dbl-like oncogene (Dbs) and intersectin are primarily involved in Cdc42 [65, 66]. These GEFs demonstrate remarkable specificity in recognizing and binding to their respective Rho-GTPases, enabling precise spatial and temporal regulation. Significantly, the activation mechanism of GEFs differs fundamentally from that of cell surface receptors. Upon extracellular stimulation, surface receptors engage with their cognate ligands, initiating signaling cascades that ultimately activate GEFs, which in turn promote Rho-GTPase activation, rather than directly activating Rho proteins. Each individual GEF exhibits specific recognition features for individual members of the G protein family. Typically, there is no cross-reactivity between members of different G protein families. Similarly, these rules also apply to GAPs.

The regulatory network of Rho-GTPases also involves negative regulators, particularly guanine nucleotide dissociation inhibitors (GDIs). GDIs maintain Rho-GTPases in an inactive state by stabilizing the Rho-GDP complex, preventing GTP exchange and subsequent activation [67]. In keratinocytes, the interaction between Cdc42 and GDI has been found to play a significant role in stabilizing junctions between adjacent epithelial cells. The underlying mechanism is that GDI prevents subsequent GTP loading, thereby ensuring cellular stability and the integrity of the skin tissue barrier. Widely distributed in cellular compartments, GDI forms complexes with various Rho proteins, effectively suppressing their activities. Mechanistically, GDI is constrained by its nonspecific association with the switch region of Rho-GTPases. Subsequently, electrostatic interactions drive the specific binding between the polybasic region at the carboxyl terminus of Rho-GTPases and two distinct negatively charged clusters on GDI1. This interaction, coupled with geranylgeranylation, leads to membrane dissociation and nonspecific displacement, thereby preventing the activation process [68]. Another class of negative regulators, GAPs, function by accelerating GTP hydrolysis. GAPs inhibit Rho activity by enhancing the intrinsic GTPase activity of Rho proteins, converting GTP to GDP and thereby inactivating Rho proteins. The regulatory mechanisms governing Rho-GTPases are remarkably complex and multifaceted, involving not only GEFs, GAPs, and GDIs but also cell surface receptor-mediated signaling, transcriptional regulation, post-translational modifications, and protein–protein interactions [69, 70]. Furthermore, the upstream signaling pathways regulating Rho-GTPase encompass various cytokines and growth factors, among others. These extracellular signaling molecules bind to surface receptors on host cell, initiating signaling cascades that ultimately modulate Rho protein activation. The integration of these diverse regulatory mechanisms allows cells to precisely control Rho-GTPase activity in response to various intracellular and extracellular signals, including viral infections [71].

### Viral modulation of Rho-GTPase downstream signaling pathways

The regulation of Rho-GTPase activity during viral infection represents a critical balance between activation and inactivation states, which is essential for successful viral invasion and transmission at both cellular and organismal level. Upon activation, Rho proteins orchestrate cytoskeletal reorganization, membrane dynamic, and intracellular transport processes through their downstream regulators, creating a cellular environment conducive to viral infection. One of the best-characterized Rho-mediated pathways in viral infection is the Rho/ROCK signaling cascade. Activated Rho protein interacts with ROCK, stimulating its kinase activity [50], which subsequently initiates a phosphorylation cascade involving downstream targets such as myosin light chain (MLC) and LIM kinase (LIMK) [72, 73]. This signaling pathway promotes cytoskeletal contraction and reorganization, facilitating viral particle transport to the cell membrane and enhancing virus–cell membrane fusion, thereby increasing viral infectivity [74]. Beyond cytoskeletal remodeling, ROCK also modulates viral infection through regulation of intracellular transport mechanisms [75]. Specifically, ROCK-mediated ADP-ribosylation factor 6 (Arf6) facilitate the formation and endocytic vesicles of transport. The ROCK-Arf6–cytoskeleton–transport vesicle signaling cascade is critically involved in mediating transmembrane transport mechanisms [76], which serve as crucial conduits for viral entry into host cells, thereby promoting viral replication and propagation [77]. Another essential downstream effector of the Rho signaling is mDia, which plays a significant role in viral infection by regulating actin polymerization and stress fiber formation [51]. Through its ability to promote MT formation and stabilization at the cell membrane, mDia creates a transport channel for viral entry and intracellular movement [78]. The Rho-PAK signaling axis represents another important pathway in viral infection, regulating cellular migration, adhesion, and proliferation through the phosphorylation of downstream substrates including v-crk sarcoma virus CT10 oncogene homolog (Crk) and Crk SH3 domain-binding guanine nucleotide exchange factor (C3G) [79, 80]. These cellular alterations significantly impact viral dissemination and replication within cells [81]. Another important point to mention is that PAK can also influence viral infection by regulating intracellular signaling pathways. PAK-mediated signaling extends beyond cytoskeletal regulation, influencing viral infection through modulation of intracellular signaling pathways [82]. For instance, PAK activation of the mitogen-activated protein kinase (MAPK) pathway can stimulate the expression and release of inflammatory factors, which may subsequently affect viral replication and transmission dynamics [83].

The collective impact of Rho protein activation on viral infection efficiency is well documented, with multiple mechanisms contributing to enhanced viral success. Cytoskeletal reorganization, particularly actin polymerization and rearrangement, creates favorable pathways for viral transport and release, thereby increasing virus–host cell interactions [28, 84]. Moreover, certain viruses, such as Zika virus (ZIKV), can exploit host cell interaction mechanisms and hijack the cytoskeleton to compromise the integrity of the blood–testis barrier (BSB) [85]. Indeed, beyond facilitating viral infection, the downstream pathways of Rho-GTPases activation are also implicated in the modulation of immune responses. A study on host defense peptide analogs demonstrated that they can independently mediate the production of inflammatory cytokines and the recruitment of leukocytes, independent of the Rho-GTPases signaling cascade, while exerting a negative regulatory role in suppressing inflammatory cytokine production [86, 87]. Consequently, these peptides represent a promising class of potential therapeutic targets in cases of hyperinflammatory responses, particularly in lethal cytokine storms induced by coronavirus infections [88].

### Interplay between Rho-GTPases and other signaling pathways

**Fig. 2:**
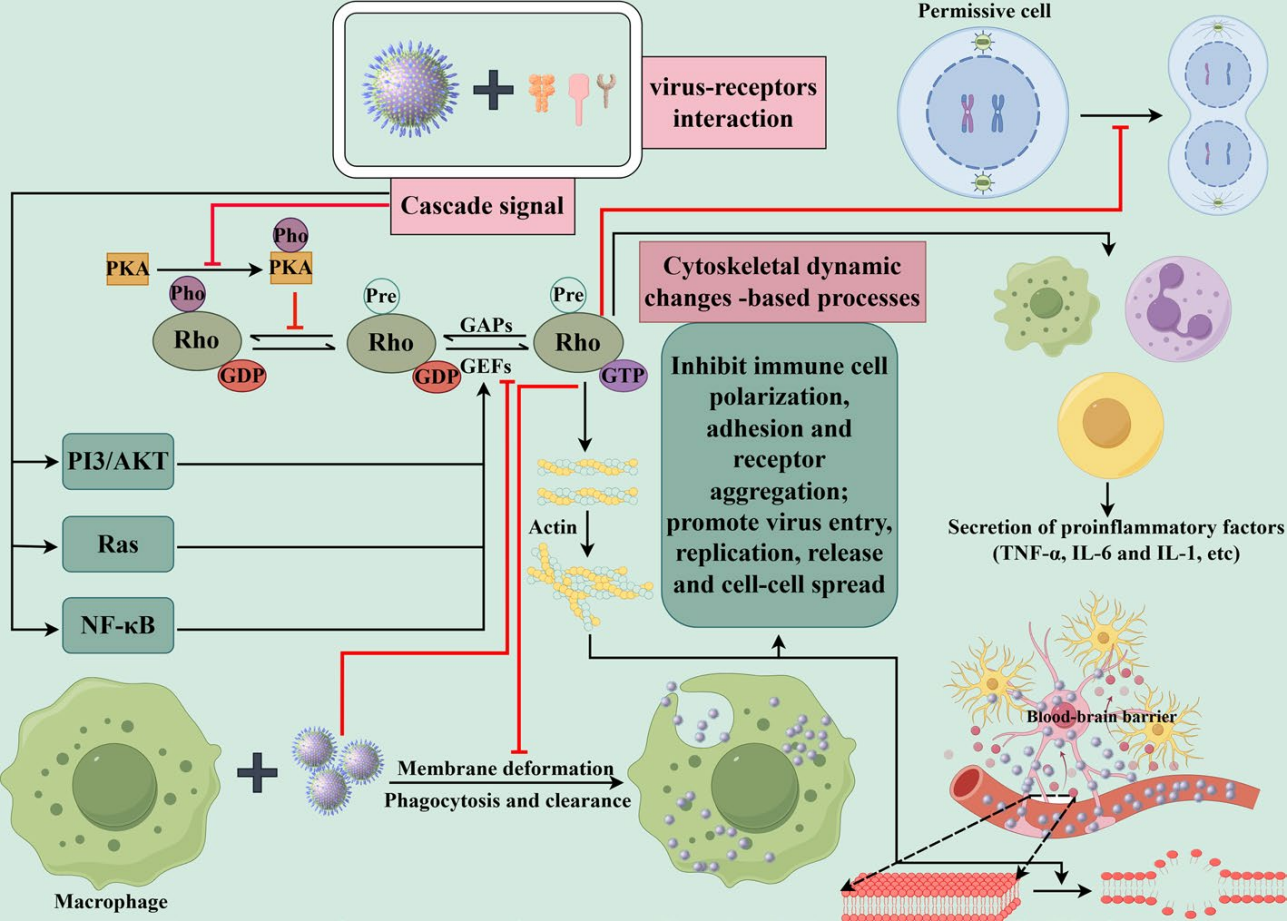
Model diagram illustrating the interaction of Rho subfamily proteins with key signaling pathways during viral infection. Upon viral binding to membrane surface receptors, a cascade of signaling events is initiated, leading to Rho protein activation through multiple pathways including PI3K/AKT, Ras, and NF-κB. Concurrently, Rho proteins suppressed by upstream regulatory molecules (e.g., PKA) can be activated through these signaling cascades. These intricate interactions ultimately converge on cytoskeletal rearrangement, which is essential for multiple stages of the viral life cycle, including viral entry, intracellular transport, replication, and subsequent release and transmission. Notably, virus-mediated manipulation of Rho activation through cytoskeletal modulation serves multiple pathogenic functions: (1) suppression of host cell proliferation and inhibition of immune cell-mediated virion phagocytosis, (2) induction of pro-inflammatory factor release from immune cells, thereby facilitating viral spread, and (3) modulation of cellular barrier permeability, particularly evident in viral penetration of the blood–brain barrier and respiratory tract epithelia. This latter function is mediated through Rho-cytoskeleton-dependent regulation of local cell permeability, creating favorable conditions for both physical pathogen translocation and establishment of local infection foci. The coordinated regulation of these processes through Rho-cytoskeleton interactions highlights the sophisticated mechanisms by which viruses exploit host cell signaling networks to establish and propagate infection, while simultaneously evading host defense mechanisms

During viral infection, host cells activate a cascade of signal transduction mechanisms to counteract viral invasion. Among these, Rho proteins, key regulators of cellular signaling pathways, play a pivotal role in multiple stages of viral infection. They not only directly mediate critical processes such as viral entry, replication, and release but also interact with other signaling pathways to coordinately regulate the cellular immune responses (Fig. 2).

**PI3K/Akt signaling pathway**: The PI3K/Akt signaling pathway is a crucial cellular signaling pathway involved in regulating processes such as cell survival, proliferation, and migration [89]. From the perspective of the Rho protein, it is conceivable that the Rho protein in host cells may directly or indirectly regulate the PI3K/Akt signaling pathway. On one hand, the Rho protein may be directly involved in the activation process of PI3K, thereby impacting its phosphorylation [90]. The activation of Rho-GTPases in social amoeba cells leads to the phosphorylation of AKT by mammalian target of rapamycin complex 2 (mTORC2), ensuring cytoskeleton-dependent directional cell migration. On the other hand, the Rho protein may also indirectly influence the activity of the PI3K/Akt signaling pathway by regulating downstream target proteins (such as PAKs, ROCK, etc.) [28, 91]. Similarly, the PI3K/Akt signaling pathway can have an impact on the activity of the Rho protein. As a downstream target kinase of PI3K, Akt has the potential to regulate Rho protein activity through phosphorylation [92]. The increase in RhoA-GTP benefits from the phosphorylation of AKT and shows a significant correlation in positive cancer cell lines. Additionally, the PI3K/Akt signaling pathway may indirectly regulate Rho protein activity by influencing other pathways, such as the MAPK pathway. This physiological process, mediated by adaptor molecules, ensures the regulation of cytoskeleton-mediated cell motility [93]. Similarly, the study by Cuartas-Lopez et al. confirmed the importance of the interaction between Rho-GTPases and the PI3K/Akt signaling pathway for the establishment of DENV infection. Both the use of their inhibitors and specific knockdown experiments resulted in weakened cytoskeletal reorganization, followed by a reduction in viral infection [28]. In general, the crosstalk in this interaction may contribute to the recognition and response of host cells to viral invasion, while viruses may also exploit this interaction to facilitate their infection and replication [36].

**Ras signaling pathway**: The Ras signaling pathway represents a critical intracellular signaling mechanism that plays a pivotal role in regulating diverse biological processes, including cell proliferation, differentiation, and programmed cell death (apoptosis) [94]. As members of the small GTPase protein family, Rho and Ras share significant structural and functional similarities, with extensive crosstalk in their molecular mechanisms and biological roles [95]. Both pathways are critically implicated in various cellular processes and disease states, including viral infections, highlighting their interconnected regulatory networks [78, 96]. The Rho protein modulates the Ras signaling pathway through multiple molecular mechanisms, exerting regulatory control over its activity and downstream effects [35]. One such mechanism involves its interaction with the Ras protein or its downstream molecules, thereby influencing the transmission and effects of Ras signals [97]. For instance, the RhoA protein possesses the ability to potentiate Ras activity through the activation of p115-labeled Rho guanine nucleotide exchange factor (p115RhoGEF) or by directly binding to Ras, thereby facilitating cell proliferation and survival [98, 99]. Additionally, the Rho protein indirectly impacts the Ras signaling pathway by modulating the cytoskeleton and membrane fluidity, which facilitates virus invasion and replication, such as adenovirus (Ad) [100]. Indeed, the activation of the Ras signaling pathway can also modulate the activity state of Rho proteins by influencing their upstream regulatory factors or downstream effector molecules. For instance, specific kinases within the Ras signaling pathway are capable of phosphorylating GDI, which reduces its binding affinity for Rho proteins. This phosphorylation event facilitates the transition of Rho proteins from an inactive state to an active state, thereby promoting their functional activation [101]. Moreover, it is crucial to emphasize that the Ras signaling pathway exerts an indirect impact on the modulation of Rho protein activity by modulating intracellular redox status or calcium ion concentrations [102, 103]. Fundamentally, the crosstalk between the Rho and Ras signaling pathways plays a critical role in viral infection by facilitating viral entry into host cells and enhancing viral replication efficiency [35]. Rho proteins and the Ras signaling pathways regulate cytoskeletal dynamics and membrane remodeling, activating cell signaling pathways that promote viral replication and establishing a cellular environment conducive to viral invasion and propagation [28]. Targeting Rho kinase through genetic or chemical inhibition not only blocks viral infection but also suppresses the intracellular trafficking of viral particles, including their nuclear import and export. These experimental observations have been demonstrated during IAV infection in human umbilical vein endothelial cells (HUVECs) and Madin–Darby canine kidney cells (MDDCs), with the core mechanism involving the disruption of cytoskeletal remodeling [104].

**NF-κB signaling pathway**: NF-κB is a protein complex present in virtually all animal cell types and is involved in cellular responses to stimuli such as cytokines, free radicals, and bacterial and viral antigens, especially in regulating infection-induced immune responses [105]. The crux of the interaction between the Rho protein and NF-κB signaling pathway lies in their reciprocal modulation and synergistic interplay in immune response and inflammatory cascade [106, 107]. The dynamic interplay between these elements is primarily characterized by the phosphorylation and subsequent degradation of Rho-GTPase through direct interaction with the inhibitory protein inhibitor of nuclear factor kappa-B alpha (IκBα). This process facilitates the release and nuclear translocation of NF-κB, leading to its transcriptional activation [108, 109]. Concurrently, Rho-GTPase indirectly promotes NF-κB activation by enhancing the downstream MAPK signaling pathway [108]. Importantly, this regulatory effect is especially prominent during the early stage of viral infections [28], enabling the swift activation of the host cell’s immune response [110]. However, the regulation of NF-κB by the Rho signaling pathway primarily occurs at the transcriptional level [111]. Following viral infection, NF-κB is activated and translocated into the nucleus to enhance Rho protein transcription by binding to the gene promoter region [57]. Significantly, the incidence of these phenomena is particularly conspicuous in the advanced stages of viral infection, amplifying the host cell response to virus-induced damage by upregulating the expression of Rho proteins [112]. In brief, the Rho protein promotes immune and inflammatory responses in host cells by modulating the NF-κB signaling pathway, thereby effectively inhibiting viral replication and spread [113, 114]. Concurrently, the NF-κB signaling pathway enhances self-repair and anti-apoptotic capabilities of host cells through the transcriptional and post-transcriptional regulation of Rho protein expression, alleviating cellular damage induced by viral infection [115].

**Cytoskeleton and cell motility-related signaling pathway**: The cytoskeleton is a complex network composed of microfilaments, MTs, and intermediate filaments, providing mechanical support to cells and actively participating in critical biological processes, including organelle positioning, material transport, and signal transduction [116]. Upon binding to specific cell surface receptors, the virus initiates cytoskeletal reorganization through precise molecular signaling, resulting in structural changes to microfilaments and MTs that promote viral endocytosis viral endocytosis [117]. Both knockdown and overexpression experiments, as well as pharmacological inhibitors and RNA interference studies, have confirmed the significant correlation between the Rho-cytoskeleton and viral infection. Among these, the most representative is the driving force exemplified by molecular motors, which facilitates the intracellular and extracellular transport processes mediated by the Rho superfamily. At its core, this phenomenon is attributed to the enhancement of viral endocytosis and cellular motility, which collectively promote viral migration, replication, and release within the host cell [118]. In particular, RhoA, Rac, and Cdc42 are pivotal in regulating cell motility and migration by activating downstream effector proteins such as ROCK, PAK, and WASP [5, 12, 13]. These signaling pathways are essential for viral tropism, replication, and dissemination within the intracellular environment [119]. Collectively, the interplay among Rho protein, cytoskeleton, and cell motility signaling pathways during viral infection represents a multifaceted process [120]. These interactions not only facilitate viral dissemination and replication within the host cell but also elucidate the biological mechanisms underlying the host cell’s response to viral infection [59].

**Immune response-related signaling pathways**: The immune system, as the body’s defense barrier, recognizes viruses through pattern recognition receptors (PRRs) and initiates immune response to facilitate viral clearance [121]. During the viral life cycle, Rho proteins play a critical role in cell migration, invasion, and the modulation of antiviral immune response [4, 122]. They regulates the proliferation and function of immune cells while simultaneously impairing their ability to recognize and eliminate viruses, thereby facilitating the spread of infected cells to surrounding tissues and expanding infection scope [123]. More specifically, Rho proteins modulate the migration and adhesion of immune cells by regulating cytoskeletal remodeling and cell motility [4]. In certain contexts, Rho activation may inhibit the proliferation and T or B cell receptor (TCR/BCR)-mediated signal transduction for the activation of T cells and B cells, thereby reducing their capacity to recognize and clear viruses [124, 125]. Additionally, Rho proteins may also impair the capacity of immune cells to release immune effector molecules, such as cytokines and antibodies, ultimately weakening the efficacy of antiviral immune responses [112, 126]. Of equal significance, excessive inflammatory response can exacerbate viral infections [127]. Of note, Rho-GTPases are integral in regulating the progression of viral infection by modulating inflammatory processes [128]. Specifically, an overactive inflammatory can worsen viral infection through Rho-GTPase activation, which stimulates the production and release of pro-inflammatory cytokines, including interleukin (IL) [129], interferon (IFN) [130], and tumor necrosis factor (TNF) [112], thereby amplifying inflammation and promoting viral replication. Another critical aspect is the potential of Rho-mediated immune responses to stimulate cell proliferation and inhibit apoptosis, creating a favorable environment for viral replication. This is mediated through downstream effector interactions with cytoskeletal proteins and key cell cycle regulatory points following Rho activation [131, 132]. Conversely, when the inflammatory response mobilizes antimicrobial proteins to directly eliminate pathogens, cell proliferation is inhibited and apoptosis is promoted [133]. Alternatively, during the adaptive immune phase, infected cells are targeted by T cells and B cells, which either initiate a pathogen-specific immune response or directly inhibit the cell cycle to prevent an increase in infected cell numbers [134]. Indisputably, the interplay between Rho-GTPases and other pathways constitutes a complex regulatory network that critically influences every stage of viral infection. These patterns highlight the complexity of viral pathogenesis and offer valuable insights for the development of innovative antiviral therapeutics and treatment strategies [135].

## Advances in understanding the role of Rho in specific viral infections

### Case study: IAV infection

The involvement of Rho protein in viral infection is intricate and multifaceted, characterized by a relatively limited yet specific set of functions within the infection process [4]. Consistent with the general viral infection process, IAV, as an enveloped RNA, enters susceptible cells virus via endocytosis. In this context, Rho proteins leverage their function as molecular switches to regulate intracellular signaling pathways implicated in viral infection [136]. Specifically, the activation status of Rho protein directly affects the expression and activity of signaling molecules associated with IAV infection, such as intracellular kinases and phosphatases [104, 137]. Similarly, the Rho protein-mediated regulation of the cytoskeleton is indispensable for the invasion and replication of the IAV as well [138]. By modulating cell microtubule, microfilament, and intermediate fiber structures, the Rho proteins directly promotes IAV migration, attachment, and replication processes [138]. For instance, RhoA/Rho kinase activation induces MLC phosphorylation, driving actin cytoskeleton remodeling. This process is characterized by stress fiber formation, focal adhesion complex assembly, and pseudopodia formation, ultimately enhancing IAV proliferation. Conversely, when the signaling cascade is inhibited through genetic or chemical interventions, the efficiency of viral infection, including attachment and entry on cell surfaces, particularly nuclear transport, is significantly reduced [104, 139]. It is imperative to emphasize that the Rho protein is fundamentally involved in modulating endothelial cell barrier function, and alterations in its permeability can profoundly promote IAV infection and transmission [104]. Excessive Rho activation destroys endothelial cell permeability by enhancing the activity of myosin-based contractile components and interendothelial junctions, which is hijacked by IAV as a mechanism for ensuring transmission and diffusion within the host [104]. On the basis of the potential functions described above, although direct evidence regarding the specific role of Rho proteins in IAV infection remains limited, we hypothesize that Rho proteins may play a critical role in IAV infection by modulating these pathways, facilitating cytoskeletal reorganization, and regulating endothelial cell barrier permeability [48, 140].

### Case study: herpesvirus infection

Herpesviruses are DNA virus characterized by their complex structure and unique ability to establish latent infections. They exhibit a high prevalence in the human population and demonstrate a remarkable neurotropism, enabling them to target the brain and establish lifelong latency with the potential for reactivation [141]. Rho-GTPases, functioning as intracellular signaling transducers, exert diverse physiological roles and are intricately involved in the entire herpesvirus infection process [20]. During the invasion stage, Rho protein facilitates the fusion of the viral envelope with the cell membrane by modulating the dynamic changes [57]. Specifically, Rho proteins change the microtubule cytoskeleton architecture of the cell membrane through the activation of downstream effector molecules such as ROCK, enhancing membrane pliability and deformability, thereby promoting viral envelope–cell membrane fusion [142]. Meanwhile, promotion of lamellipodia formation and dynamic stress fiber assembly, which has been observed in primary chicken embryo skin cells (CESC) infected with Marek’s disease virus (MDV), is critically important for cell-to-cell viral spread. Following viral entry, Rho proteins regulate the intracellular transport of viruses by modulating cytoskeletal components, including MTs and actin filaments [143]. For example, RhoA activates downstream effectors such as mDia to promote actin polymerization and reorganization, providing the mechanical force necessary for viral transport. One of the most representative examples is the invasion of neuronal cells by herpes simplex virus type 1 (HSV-1). Upon successful entry, HSV-1 traffics between epithelial cells, the trigeminal nerve, and the brain, enabling the transition between lytic infection and latent infection under host immune pressure. Simultaneously, Rho proteins also influence the vesicular transport system, ensuring that viral particles are directed along the correct pathways to reach the nucleus [144]. When cells are treated with lipid raft (LR) disruptors such as methyl-β-cyclodextrin (MβCD) or nystatin, the association of internalized viral capsids with MTs and the quantity of nuclear-associated viral DNA are significantly reduced. Disorganized and disrupted MTs, along with thickened and rounded plasma membranes, are observed. These effects are most directly attributed to RhoA-GTP-mediated cytoskeletal cargo transport. During the early and middle phase, Rho-GTPases modulate viral genome replication and protein expression by regulating signal transduction and gene expression [145]. On one hand, Rho-GTPases enhance viral genes transcription and translation by activating pathways such as MAPK [146]. On the other hand, they regulate the stability and function of viral proteins through signaling cascades such as PI3K/Akt [147]. These mechanisms collectively promote efficient viral genome replication and robust expression, facilitating viral proliferation and dissemination [20]. Although the precise molecular mechanisms underlying Rho-GTPases involvement in herpesvirus remain unclear, it is hypothesized that they may indirectly influence these processes through several mechanisms. By regulating cytoskeletal dynamics, such as MT and actin filament recombination and stability, Rho-GTPases affect intracellular viral transport and localization, thereby indirectly participating in viral assembly. Additionally, Rho-GTPases may modulate membrane fluidity and stability, potentially promoting or inhibiting fusion and separation of viral particles with the cell membrane [148]. Throughout the herpesvirus lifecycle, host cells initiate immune responses to counteract viral infection, implicating Rho-GTPases in regulating immune cell migration and adhesion [149]. This modulation ultimately impacts the host cell’s ability to recognize and clear the virus, highlighting the complex interplay between Rho-GTPases and the host immune response during herpesvirus infection.

### Case study: HIV infection

Human immunodeficiency virus (HIV) is a retrovirus that targets the immune system, primarily attacking and depleting CD4^+^ T cells, resulting in the progressive collapse of immune function and the development of acquired immunodeficiency syndrome (AIDS) [150]. During the initial phase of infection, HIV binds to CD4 receptors and co-receptors, such as chemokine (C–C motif) receptor 5 (CCR5) or chemokine (C–X–C motif) receptor 4 (CXCR4), on the host cell surface via the gp120 protein, initiating the viral infection cascade [151]. At this stage, Rho subfamily proteins such as RhoA regulate actin polymerization and depolymerization, thereby modulating the recognition and binding process between HIV and host cells [152]. Upon entry, HIV RNA is reverse transcribed into DNA by reverse transcriptase and subsequently integrated into the host genome, forming a provirus [153]. This integration is followed by proviral gene expression and the induction of host cell cycle responses in susceptible cells [154]. The provirus is then transcribed into viral RNA, which is translated into viral proteins by host ribosomes [155]. Notably, Cdc42 facilitate an optimal environment for viral replication and assembly by orchestrating cytoskeletal reorganization and cellular morphological alterations [156]. Furthermore, RhoA modulates the stability and function of HIV protein by regulating intracellular post-translational modifications, including phosphorylation and ubiquitination [157]. These regulatory mechanisms influence HIV gene expression and protein synthesis by altering host cell transcription and translation processes [158]. During the late stages of the viral lifecycle, HIV structural proteins and enzymes of HIV assemble into viral particles within host cells, which are subsequently released through budding from the host cell membrane [61]. A representative example is the HIV structural protein Tat, which significantly enhances the activation of RhoA and its downstream effectors, such as myosin phosphatase target subunit 1 (MYPT) and MLC. It also markedly upregulates the promoter activity and expression of P-glycoprotein (P-gp). These molecular events induce frequent actin polymerization and depolymerization, as well as enhance efflux function, ultimately determining the efficiency of viral particle release in human cortical cells [158]. Similarly, Rac1 has been demonstrated to facilitate the shedding of viral particles from the T cell membrane by modulating microtubule dynamics, and contributing to membrane anchoring of Gag proteins and subsequent viral particle production [61]. In brief, the response pattern of Rho proteins during HIV infection constitutes a highly sophisticated and regulatory dynamic process. These proteins are pivotal in regulating critical biological processes, including cytoskeletal reorganization, cell motility, and apoptosis, during HIV infection [152]. Their involvement underscores the complex interplay between host cellular machinery and viral replication, highlighting the multifaceted nature of HIV pathogenesis.

### Case study: RSV infection

RSV is an enveloped, single-stranded, negative-sense RNA virus that poses a significant pathogenic threat to infants and young children, especially premature infants, low-birth-weight infants, and infants with chronic diseases. It primarily proliferates in the nasopharyngeal epithelial cells of infected individuals [159]. Studies have indicated that RSV infection triggers the activation of Rho proteins, particularly RhoA. This activation is likely mediated by interactions between specific RSV viral proteins and host cell signaling molecules [160]. RSV enhances virion entry and release by modulating cytoskeletal reorganization, primarily through downstream signaling molecules such as hyperactivated ROCK and RhoA. These molecules inhibit actin fiber formation and contraction, thus creating an environment conducive for infection [122]. Moreover, RSV infection induces abnormal migration and adhesion of airway epithelial cells. Rho protein regulate these cellular behaviors behavior during RSV by modulating the PKA signaling pathway, which is associated with cell migration and adhesion, ultimately promoting viral dissemination [161]. It is noteworthy that Rho proteins orchestrate the aggregation and disaggregation of the cytoskeleton, thereby modulating cell morphology and regulating cell motility. This regulatory function also facilitates the migration and infiltration of inflammatory cells. Consequently, these mechanisms have spurred the development of small-molecule inhibitors as a promising therapeutic trend [162]. Ken Iesato and colleagues have proposed that tiotropium bromide can reduce the frequency of acute exacerbations in patients with chronic obstructive pulmonary disease (COPD) and airway inflammation. Furthermore, evidence regarding the replication of RSV suggests that the reduction of viral syncytia, the attenuation of RhoA activation, and the decrease in intercellular adhesion molecule 1 (ICAM-1) levels are the primary factors leading to the cytoskeleton-related suppression of IL-6 and IL-8 production [163]. A range of compounds are under evaluation, including biphenyl analogs, 2-5A-antisense oligonucleotides, RhoA-derived peptides, and compounds with currently unknown mechanisms of action, such as benzothiophene derivatives [164]. Additionally, targets of interest include the activation of the Rho-GTPase signaling network, which drives changes in the lipid composition and properties of filamentous actin-induced lipid microdomains. These changes are critical for determining the functionality of the assembly machinery during RSV particle assembly [165].

### Role of Rho-GTPases in other viral infections

In virology, the dynamic interplay between viruses and host cells remains a central research focus. Beyond well-studied pathogens such as IAV, HIV, herpesvirus, and RSV, numerous other viruses have been shown to intricately manipulate the Rho-GTPase signaling pathway [4, 128, 148, 157, 160]. A notable example is the infectious spleen and kidney vaccinia virus (ISKNV), which poses a significant threat to the aquaculture industry [166]. Studies have demonstrated that ISKNV significantly upregulates mRNA transcription and protein expression of RhoA and ROCK following host cell infection [167]. Further investigations revealed that inhibiting the RhoA–ROCK signaling pathway markedly suppresses the viral life cycle [78]. Similarly, avian reovirus (ARV) has been shown to induces apoptosis, autophagy, and cell fusion through its interaction with Rho proteins [168]. In Vero cells, ARV infection triggers the RhoA/Rac1 signaling pathway, leading to a significant increase in RhoA-GTP, Rac1-GTP, and NF-κB activation, ultimately accelerating syncytial formation [169]. Additionally, porcine sapovirus (PSaV) infection has been found to activate the activation of the RhoA/ROCK1/MLC signaling pathway in LLC-PK cells, and the use of specific inhibitors effectively attenuates viral protein biosynthesis, genome amplification, and virion progeny, thereby inhibiting infection progression [60]. These viruses exploit host cell Rho-GTPase signaling pathways during infection [4], functioning as critical molecular switches in physiological processes, including cytoskeleton dynamics, migration, proliferation, and apoptosis through direct or indirect interactions with viral proteins [4, 16, 148].

## Conclusions

Antiviral medications have consistently been pivotal in the prevention, management, and treatment of viral diseases. Recently, with the deepening understanding of virus–host cell interactions, Rho proteins have emerged as central regulators of intracellular signaling, sparking growing interest in their potential as targets for antiviral drugs. Studies utilizing three-dimensional (3D) models of primary cell lines and organoids for antiviral drug screening have demonstrated consistent and expected results. Furthermore, a high degree of concordance has been observed between laboratory-adapted viral strains and patient-derived viral isolates [5, 170]. Given the potential of diverse viruses to exploit or disrupt Rho-GTPase signaling cascades during infection, pharmacological agents targeting these proteins may offer broad-spectrum antiviral effects by inhibiting key stages of viral replication and dissemination [28, 126, 159, 160]. More importantly, modulation of the Rho protein signaling pathways can influence cytoskeletal remodeling, cell migration, and apoptosis to mitigate viral-induced damage to host cells [25, 96]. This has the potential to alleviate inflammation and disease symptoms caused by viral infections, ultimately improving the survival rate and quality of life of patients.

Currently, researchers have successfully identified several key molecules of Rho proteins or associated signaling pathways that are closely linked to viral infection. These molecules play a critical role in the progression of viral infection and represent promising targets for antiviral drug development [91, 96, 101, 130]. Through in vitro experiments and validation in animal models, researchers have demonstrated that drug compounds targeting these molecules exhibit significant antiviral efficacy [2, 4, 5, 171] (Table 1). By leveraging computer-aided drug design (CADD) and structural biology approaches, drug molecules can be precisely engineered to bind and inhibit these targets with high specificity [172–174]. Concurrently, iterative optimization of molecular structure and activity can enhance therapeutic efficacy while reducing off-target effects [175]. Computer-aided drug screening has been conducted through molecular docking, molecular dynamics (MD), and structure–activity relationship (SAR) analysis, focusing on Food and Drug Administration (FDA)-approved drugs, including simeprevir, ergotamine, bromocriptine, and tadalafil. The evaluation was based on binding energy, docking scores, and noncovalent interactions at receptor binding sites. Pattern recognition, structural similarity, and binding energy predictions were performed, and a multiple linear regression (MLR) model was employed for quantitative structure–activity relationship (QSAR) analysis, achieving an effective accuracy rate of over 82%. However, the multifunctional nature of Rho proteins within cells poses a significant challenge: ensuring that drugs selectively target the required virus-associated Rho proteins without disrupting the normal function of the host cell and or inducing adverse effects [37]. This necessitates a comprehensive evaluation of drug–target binding kinetics and safety profiles during the design phase, along with the implementation of strategies to improve specificity and minimize side effects [176]. Key approaches include optimizing compound–target affinity while minimizing interactions with mutated binding pockets. Furthermore, emerging approaches in drug design, such as virtual screening, targeted protein/RNA degradation, and drug resistance analysis, have been utilized to mitigate the emergence of drug resistance. Additionally, it is crucial to consider the potential for antiviral drug resistance due to prolonged usage, which could compromise therapeutic efficacy over time [177]. Therefore, designing antiviral drugs with a low propensity for resistance remains a pressing challenge that requires urgent attention.

**Table 1:** Summary of chemical drugs targeting Rho protein as antiviral targets

Rho target	Antiviral compound	Virus type	Functional mechanism	Ref.
RhoA	Bafilomycin A(1)	RSV	Restriction of viral egress and excretion of pro-inflammatory cytokines (in vitro)	[178]
Cdc42	PIK-24	RSV	Inhibition of cell-to-cell fusion during syncytium formation (in vitro)	[179]
RhoA/ROCK	HA-1077	Ebola virus (EBOV)	Reversal of the vascular permeability defects (in vivo and in vitro)	[180]
RAC1	Statin	RSV	Combination of cholesterol- and isoprenoid-mediated effects on RAC1 activation (in vitro)	[5]
RhoA/CDC42	Ivermectin and atorvastatin	COVID-19	Interference of nuclear transport and vesicle transport (in vitro)	[181]
Cdc42	ZCL278	Junin virus (JUNV)	Prevention of cellular entry of enveloped viruses (in vivo and in vitro)	[182]
RhoA	Simvastatin	IAV	Suppression of RhoA activation and LC3 membrane localization (in vitro)	[183]
ROCK1	GSK269962A	Human enterovirus A71 (EV‐A71)	Inhibition of ROCK1 kinase activity (in vitro)	[184]
Rac1	NSC23766	HSV-1	Inhibition of Rac1 activity (in vitro)	[185]
Rac1	NSC23766	IAV	Effect on activity of viral polymerase complex (in vitro)	[186]
ROCK1	Thiazovivin	Buffalopox virus (BPXV)	Induction of viral mRNA attenuation in BPXV infected cells (in vitro)	[4]
ROCK1/2	Ki16425	Severe acute respiratory syndrome coronavirus 2 (SARS-CoV-2)	Promotion of antiviral innate immunity (in vitro)	[187]
ROCK1	EV‐A71	Hepatitis C virus (HCV)	Occupancy of the activation potential involved on the surface of the ROCK1 active pocket; blocking the secretion of proinflammatory factors (in vitro)	[188]
RhoA, Cdc42, and Rac1	Atorvastatin	SARS-CoV-2	Inhibition of actin cytoskeleton-dependent trafficking (in vivo and in vitro)	[181]
ROCK	Y27632	Human cytomegalovirus (HCMV)	Inhibition of nuclear translocation and subsequent activation of ROCK	[75]
ROCK	HA-1077	Hepatitis C virus (HCV)	Inhibition of ROCK activity (in vivo and in vitro)	[189]

From a research technology perspective, virtual screening methods based on molecular docking have emerged as a critical tool for identifying novel Rho protein inhibitors [178–180]. Among these, the most commonly used are targeted protease inhibitor libraries and protein–protein interaction inhibitor libraries, which employ ligand-based and structure-based approaches, along with various filtering steps using molecular descriptors, to generate extensive final libraries. However, structure-based drug design (SBDD) represents a critical endeavor in the field of structural bioinformatics. Traditionally, this process relies on laboratory experiments to construct ligand libraries, where molecules are tested to determine their binding efficacy to protein targets. This approach is often time-consuming and costly. With the advent of supercomputers and advancements in computational power, the search for suitable ligand molecules targeting specific proteins allows for the rapid screening of potential drug candidates that interact with Rho-GTPases from extensive compound pools, significantly reducing experimental cost. Moreover, structural biology has demonstrated its profound impact on elucidating the structures and functions of the vast majority of viral proteins, leading to the development of highly effective inhibitors for SARS-CoV-2, such as Pfizer’s PF-07321332 (Paxlovid), Merck’s nucleotide inhibitor molnupiravir (Lagevrio), and the oral drug candidate VV11 [181]. However, the structural properties of Rho proteins pose substantial challenges for drug development owing to their spherical structure and strong binding affinity with substrate GDP/GTP [37]. Traditional drug discovery methods have proven inadequate for identifying effective Rho-GTPases inhibitors. Currently, only a limited number of small-molecule inhibitors targeting Rho-GTPases have been reported, many of which exhibit suboptimal activity and selectivity [157, 182], and none have advanced to clinical practice. To address these limitations, researchers are actively exploring innovative strategies. For instance, in-depth studies on the conformational regulation mechanism of Rho proteins have revealed a novel functional pocket capable of binding covalent compounds, offering new opportunities for the development of targeted inhibitors [183]. During the modeling of key structural domains or the simulation and resolution of crystal structures, variations and stability in tertiary structures, as well as potential post-translational modifications, also contribute to the diversity of target selection.

With the rapid advancement of drug design and screening technologies, such as high-throughput screening based on structural biology and CADD, it is anticipated that more potent, low-toxicity, and highly selective inhibitors of Rho protein will be discovered in the future [174, 184]. These inhibitors are expected not only to directly target specific stages of viral infection but also to promote antiviral efficacy by enhancing RNA sensing and the interferon axis triggered by foreign cytoplasmic RNA exposure [185]. Moreover, given that viruses often exploit multiple host cell signaling pathways during infection, future studies on Rho proteins may integrate insights from other signaling pathways to develop novel strategies for multitarget synergistic therapy [186]. A most compelling example is the study by Xu et al., who investigated the anti-RSV activity of *Acorus tatarinowii* ethanol extract (containing total alkaloids, lignans, and organic acids) as antiviral chemical components and conducted in vitro and in vivo experiments to evaluate their anti-RSV activity, both individually and in combination. Histopathological staining revealed that the extract, either alone or in combination, alleviated virus-induced lung lesions in mice. However, the combination of all three components resulted in significantly more pronounced reduction of lung lesions compared with individual treatments. Plaque reduction assays demonstrated that the combined treatment exhibited far stronger antiviral activity than single agents. Additionally, reverse transcription quantitative polymerase chain reaction (RT-qPCR) and Western blot analyses indicated that the mRNA and protein expression levels of key signaling molecules in the retinoic acid-inducible gene I (RIG-I) and melanoma differentiation-associated protein 5 (MDA5) pathways in mouse macrophages were downregulated by the individual or combined active components. The rise of host-directed therapy has further intensified interest in developing Rho protein-based antiviral drugs tailored to specific virus types and infection stages [187], but identifying multiantigen antiviral-host targets is extremely difficult when complete information about the human genome/kinase set is not available. However, since viruses are unlikely to mutate to compensate for missing cellular functions, the use of host-targeting inhibitors minimizes the opportunity for the emergence of drug-resistant mutants [188, 189]. This advancement promises to make antiviral treatments more precise, effective, and safe. Indeed, resistance to certain host-directed antiviral drugs can indeed occur under specific conditions, such as prolonged selective pressure from host-targeting therapies. This pressure may provide opportunities for the virus to adapt by utilizing alternative host factors or altering its affinity for the target, thereby conferring drug resistance—a phenomenon that cannot be entirely avoided.

## Abbreviations

Rho-GTPases: Rho-GTPases Ras homolog gene family-guanosine triphosphatases
GEFs: Guanine nucleotide exchange factors
GAPs: GTPase-activating proteins
PI3K/Akt: Phosphoinositide 3-kinase/protein kinase B
Ras: Rat sarcoma
NF-κB: Nuclear factor kappa-light-chain-enhancer of activated B cells
IAV: Influenza A virus
HIV: HIV Human immunodeficiency virus
RSV: Respiratory syncytial virus
Rac: Ras-related C3 botulinum toxin substrate
Cdc42: Cell division cycle 42
Rnd: Resistance-nodulation-division
Rho BTB: Rho-related BTB domain-containing protein 1
TC10: T-cell lymphoma invasion and metastasis-inducing protein 10
TCL: T-cell leukemia/lymphoma protein
WASP: Wiskott–Aldrich syndrome protein
MRCK: MRCK Myotonic dystrophy kinase-related Cdc42-binding kinase
PAK1: P21-activated kinase 1
DENV: Dengue virus
PRV: Pseudorabies virus
RABV: Rabies virus
Cyclin D1: Cyclin-dependent kinase 4 inhibitor D1
CDK: Cyclin-dependent kinase
P27KiP1: Cyclin-dependent kinase inhibitor 27 protein/cyclin-dependent kinase inhibitor
MAVS: Mitochondrial antiviral-signaling protein
Trim31: E3 ubiquitin ligase tripartite motif-containing protein 31
Caspase-8: Cysteine-dependent aspartate-specific protease-8
cFLIP: Cellular FLICE-like inhibitory protein
Ripk1: Receptor-interacting serine/threonine-protein kinase 1
GDP: Guanosine diphosphate
MTs: Microtubules
ECM: Extracellular matrix
ROCK: Rho-associated kinase
mDia: Mammalian Diaphanous
Dia-2: Diaphanous homolog 2
Tiam: T-cell lymphoma invasion and metastasis-inducing protein 1
Vav1/Vav2: Vav 1/2 oncogene
Dbl: Diffuse B-cell lymphoma
Dbl/Dbs/ITSN: Dbl-like oncogene (Dbs) and intersectin
GDIs: Guanine nucleotide dissociation inhibitors
MLC: Myosin light chain
LIMK: LIM kinase
Arf6: ROCK-mediated ADP-ribosylation factor 6
Crk: V-crk sarcoma virus CT10 oncogene homolog
C3G: Crk SH3 domain-binding guanine nucleotide exchange factor
MAPK: Mitogen-activated protein kinase
ZIKV: Zika virus
BSB: Blood–testis barrier
mTORC2: Mammalian target of rapamycin complex 2
p115RhoGEF: P115-labeled Rho guanine nucleotide exchange factor
Ad: Adenovirus
HUVECs: Human umbilical vein endothelial cells
MDDCs: Madin–Darby canine kidney cells
IκBα: Nuclear factor kappa-B alpha
PRRs: Pattern recognition receptors
TCR/BCR: T or B cell receptor
IL: Interleukin
IFN: Interferon
TNF: Tumor necrosis factor
CESC: Chicken embryo skin cells
MDV: Marek’s disease virus
HSV-1: Herpes simplex virus type 1
LR: Lipid raft
MβCD: Methyl-β-cyclodextrin
HIV: Human immunodeficiency virus
AIDS: Acquired immunodeficiency syndrome
CCR5: Chemokine (C–C motif) receptor 5
CXCR4: Chemokine (C–X–C motif) receptor 4
MYPT: Myosin phosphatase target subunit 1
P-gp: P-glycoprotein
COPD: Chronic obstructive pulmonary disease
ICAM-1: Intercellular adhesion molecule 1
ISKNV: Infectious spleen and kidney vaccinia virus
ARV: Avian reovirus
PSaV: Porcine sapovirus
3D: Three-dimensional
CADD: Computer-aided drug design
MD: Molecular dynamics
FDA: Food and Drug Administration
SAR: Structure–activity relationship
MLR: Multiple linear regression
QSAR: Quantitative structure–activity relationship
RT-qPCR: Reverse transcription quantitative polymerase chain reaction
EBOV: Ebola virus
JUNV: Junin virus
EV‐A71: Human enterovirus A71
BPXV: Buffalopox virus
SARS-CoV-2: Severe acute respiratory syndrome coronavirus 2
HMCV: Human cytomegalovirus
HCV: Hepatitis C virus
SBDD: Structure-based drug design
RIG-I: Retinoic acid-inducible gene I
MDA5: Melanoma differentiation-associated protein 5
